# Scalp cooling to prevent chemotherapy-induced alopecia^☆^^☆☆^

**DOI:** 10.1016/j.abd.2020.03.005

**Published:** 2020-06-16

**Authors:** Giselle de Barros Silva, Kathryn Ciccolini, Aline Donati, Corina van den Hurk

**Affiliations:** a Center of Oncology, Hospital Alemão Oswaldo Cruz, São Paulo, SP, Brazil; b Department of Hematology/Oncology, Mount Sinai Hospital, New York, NY, United States of America; c Department of Dermatology, Hospital do Servidor Público Municipal de São Paulo, São Paulo, SP, Brazil; d R & D Department, Netherlands Comprehensive Cancer Organisation, Utrecht, The Netherlands

**Keywords:** Alopecia, Antineoplastic combined chemotherapy protocols, Cooling, Drug therapy, Prevention and mitigation

## Abstract

Chemotherapy-induced alopecia causes an important impact on cancer patients and its risk of persistence is currently a considerable issue in cancer survivors. Of the various interventions proposed for the prevention of chemotherapy-induced alopecia, scalp cooling has emerged as an effective and safe strategy. This paper aims to provide an overview on scalp cooling and chemotherapy-induced alopecia prevention.

## Introduction

Chemotherapy-induced alopecia (CIA) is one of the most reported unpredictable adverse events (AEs) experienced by cancer patients and survivors,^1^ with an overall incidence of 65%.^2^ It has been reported as the most disturbing condition of cancer treatment by most (88%) women receiving perioperative chemotherapy.^1^ Patients may decline life-prolonging chemotherapy to avoid developing alopecia.^1^, ^3^, ^4^ Furthermore, CIA strongly influences how others perceive cancer patients, the visibility of disease, social relationships, and sexuality.^1^, ^3^, ^5^ In addition to physical and psychosocial impairment, CIA can also be financially detrimental due to expensive cosmetic products such as wigs and hair regrowth treatments.^6^

Many strategies have been tested to minimize CIA, among which scalp cooling has proven to be the most effective.^7^, ^8^ Since recent publications on its efficacy and safety as a preventive method against CIA,^7^, ^8^, ^9^ this procedure has been increasingly employed in Brazil and worldwide. The National Comprehensive Cancer Network (NCCN) Clinical Practice Guidelines in Oncology have recently added a recommendation of scalp cooling treatment for CIA prevention (category 2A) for breast cancer patients.^10^

The present article aims to provide an overview of CIA and scalp cooling so that dermatologists can become familiar with these topics, which have become more common in clinical practice.

## Understanding chemotherapy-induced alopecia

**Figure 1:**
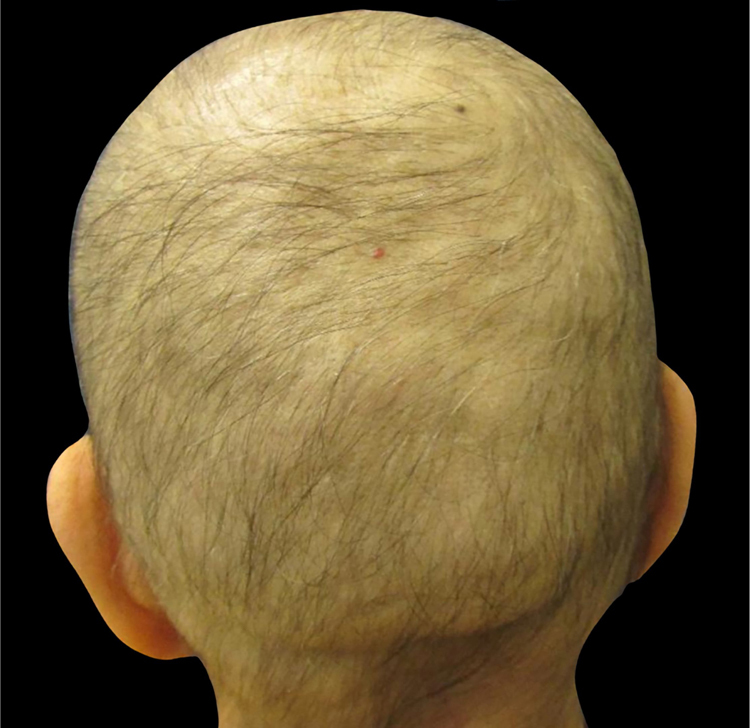
Chemotherapy-induced alopecia. Breast cancer patient at the end of the treatment with four cycles of doxorubicin and cyclophosphamide (Photo courtesy of Lívia Nicoletti Ariano).

Most chemotherapeutic agents are cytotoxic drugs that affect proliferating cancer cells. Other normally proliferating cells, *e.g.*, the hair matrix cells (in anagen phase 90% of the time) and bone marrow, are unintentional targets of chemotherapy. Patients receiving – among other drugs – anthracyclines (*i.e*., doxorubicin and epirubicin), taxanes (*i.e*., docetaxel and paclitaxel), or etoposide develop alopecia, often referred to as anagen effluvium (Fig. 1).^8^, ^11^, ^12^, ^13^, ^14^

Clinically, alopecia is most noticeable on the scalp, which has the highest density of terminal hair follicles in anagen phase, and typically appears within days to weeks after initiation of treatment with many chemotherapeutic agents.^14^ The interruption of hair follicle mitotic activity contributes to the fragility of the proximal portion of the hair shaft, and consequently breakage within the hair canal.^2^ After cessation of chemotherapy, in most cases, regrowth begins within 1–3 months; however, it can present with changes in texture, color, and/or thickness.^13^, ^14^, ^15^ Depending on the degree of hair-follicle stem-cell damage, regrowth generally takes up to six months after cessation of chemotherapy.^8^, ^14^

**Figure 2:**
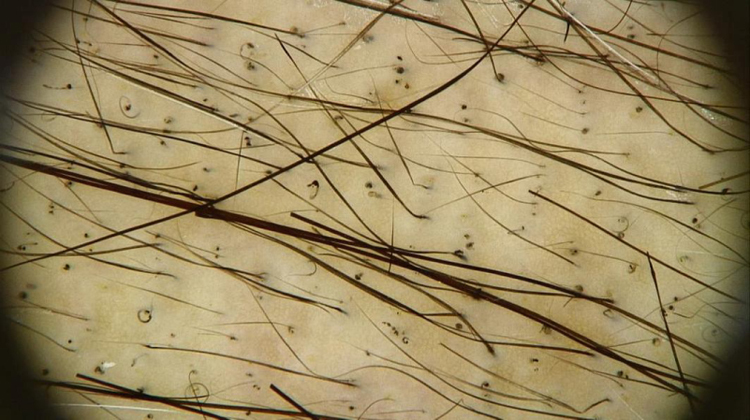
Trichoscopy of chemotherapy-induced alopecia. Trichoscopic findings in the region of the vertex of the scalp of the patient in fig. 1: multiple black dots, circular hair, and growing hair. Rare terminal hairs, some showing points of constriction (Pohl-Pinkus) in their extension, referring to previous cycles of chemotherapy (Photo courtesy of Lívia Nicoletti Ariano).

The trichoscopic findings of CIA show the changes suffered by the hair shaft throughout chemotherapy, varying according to the phase of the treatment. Broken hairs, black dots, flame hairs, and Pohl-Pinkus constrictions may be seen in the first months of chemotherapy. Additionally, regrowing hairs, rare terminal hairs, and circle hairs are usually observed at the end of the treatment (Fig. 2).^16^

Considering the type of the chemotherapeutic agent, the incidence of CIA may range from 60% to 100% with topoisomerase inhibitors (*i.e*., irinotecan, etoposide, doxorubicin), >80% with taxanes (*i.e*., docetaxel, paclitaxel), and >60% with alkylating agents (*i.e*.; cyclophosphamide, ifosfamide), whereas antimetabolites (*i.e*.; 5FU, methotrexate, gemcitabine) present a lower risk.^17^, ^18^

Besides the chemotherapy drug type, common risk factors for CIA include dose, pharmacokinetic profile, and combination regimens with various concurrent cytotoxic agents.^14^ The degree of hair loss can also vary with age, comorbidities, and nutritional/hormonal status.^14^

### Persistent chemotherapy-induced alopecia: an increasing concern

In some instances, hair loss may be persistent (persistent chemotherapy-induced alopecia, PCIA), which is defined as the absence or suboptimal hair growth persisting beyond six months after cessation of chemotherapy.^13^ The incidence of PCIA can range from 14% in childhood cancer survivors to 30% in breast cancer survivors.^19^ In the 1990s, the first reported cases of PCIA occurred after high-dose chemotherapy regimens (busulfan and cyclophosphamide) received before bone marrow transplantation.^20^ Radiation and taxane-based chemotherapy regimens have also been involved in PCIA.^21^ In a recent cohort of Asian breast cancer patients, about 42% had PCIA after three years of chemotherapy completion, with higher risk using taxanes, which appear to be more cytotoxic to hair-follicle stem cells.^15^ Relevant impact on self-image was reported in those patients.^15^, ^22^

### Strategies for chemotherapy-induced alopecia prevention

Considering the impact of CIA in cancer patients, several medications and procedures have been tested to reduce or prevent CIA. A large number of studies have used animal models, with variable and not yet clinically proven results.^23^, ^24^, ^25^, ^26^, ^27^, ^28^ A few products have been tested in humans in small studies with positive results, but more evidence is lacking, except for scalp cooling, whose safety and effectiveness have been frequently described in recent years (Table 1). Some topicals, such as minoxidil or bimatoprost, have been shown effective in stimulation of hair regrowth after chemotherapy, with no preventive effect on CIA.^29^, ^30^, ^31^, ^32^, ^33^, ^34^, ^35^, ^36^, ^37^, ^38^, ^39^, ^40^, ^41^, ^42^, ^43^, ^44^

**Table 1:** Preventive strategies for chemotherapy-induced alopecia (CIA) in animal models and in human trials

Studied in animal models	Studied in human trials: not effective	Studied in human trials: effective
Anti-doxorubicin^29^	Topical minoxidil^23^	AS101^a^^30^
Topical epinephrine and norepinephrine^31^	Parathyroid hormone receptor (PPR) ligands^32^	Scalp cooling^9^, ^33^, ^34^
Topical n-acetyl cysteine^35^	Scalp tourniquet^36^	
Topical antioxidants (resveratrol, aminothiol)^31^	Topical calcitriol^37^	
Topical cyclosporine A^38^	DHL-HisZnNA^39^	
Immunomodulators (Il-1,^40^ imuvert^41^)		
Acid FGF, EGF^40^		
CDK2 inhibitors^42^		
Caspase-3 inhibitors^43^		
Multi-target iron chelator M30^44^		

a More evidence needed.

Since the 1970s, a variety of scalp cooling techniques – such as cryogel bags, cold caps, and cooling machines – have been used for CIA prevention.^12^, ^25^, ^26^, ^27^, ^28^ Cold caps (*e.g*., Elasto-gel, Penguin) and electronic cooling machines (*e.g*., Paxman, Dignitana) are the most used worldwide.

In Brazil, three devices have approval from the Brazilian Health Regulatory Agency (Agência Nacional de Vigilância Sanitária [ANVISA]) for scalp cooling during chemotherapy: Elasto-gel, Paxman and Capelli. The latter is a device made in Brazil, using cold air, whose studies regarding its efficacy are about to be published.

### Working mechanism

The beneficial effects of scalp cooling are caused by vasoconstriction in the scalp skin, decreased intrafollicular metabolic rate, and decreased follicular drugs uptake, which in theory reduce follicular exposure to harmful cytotoxic effects at their peak plasma concentrations.^3^, ^11^, ^45^, ^46^, ^47^, ^48^

### Efficacy

The efficacy of scalp cooling was recently confirmed by Nangia et al. in a randomized-controlled trial with early-stage breast cancer patients that received at least four cycles of taxane- and/or anthracycline-based chemotherapy, in which the experimental group used a Paxman device for scalp cooling, started 30 min prior chemotherapy infusion and maintained throughout infusion and for 90 min after infusion. In the scalp cooling group, success was reported in 50.5% of women (50.5%; 95% CI 40.7–60.4%) compared to 0% in the control group (0%; 95% CI 0–7.6%).^9^ Rugo et al., in a recent systematic review and meta-analysis which included ten randomized controlled trials (two with a Paxman device and the others with cryogel caps or bag), endorsed the positive effect of scalp cooling in reducing CIA in patients with solid tumors (RR = 0.54; 95% CI 0.46–0.63; I2¼ = 11%; *p* < 0.00001).^7^ Shah et al. analyzed controlled and randomized clinical trials (CCT and RCT, respectively) evaluating scalp hypothermia for CIA prevention, with reduction in the incidence of CIA by 2.7 (in CCT) to 3.9 fold (in RCT) in scalp-cooled patients. There were 18 trials included in the review, with different scalp cooling techniques – ranging from older and less complex cooling caps to digitally controlled machines.^49^ Scalp cooling machines from Paxman and Dignitana have been approved by the Food and Drug Administration (FDA), and have been incorporated into the NCCN guidelines for breast cancer patients.

### Pre-cooling time and post-infusion cooling time

The duration of scalp cooling appears to be an influential factor in hair preservation.^50^ Cooling is generally started 30 min before infusion (pre-cooling time [PCT]) so that the scalp skin temperature has reached its lowest peak at the moment the drug enters hair follicles. Intradermal or subcutaneous scalp temperature during scalp cooling tends to be on average around 22 °C, the target temperature suggested by Gregory et al.^51^ However, low scalp temperature range may vary from one patient to another for unknown reasons. It may be due to differences in the insulating action of scalp structures (hair, dermis, and subcutaneous tissue), heat dissipation, skull anatomy, and reflex thermal reaction to vasoconstriction. Those with intradermal scalp temperature lower than 18 °C are more likely to have better hair protection with scalp cooling.^49^, ^52^

The cooling procedure is maintained during the chemotherapy infusion and kept for a period of time after its end (post-infusion cooling time [PICT]), depending on the chemotherapy regimen.

Some studies involving cold caps reported they were unable to maintain low temperatures for the entire duration of the planned treatment time. It has, however, been suggested that the caps should be changed three to four times, or every 25 min, during the chemotherapy infusion and PICT, to ensure a stable temperature.^48^ However, cooling machines with thermostat management keep a stable temperature and require fewer nursing interventions.^18^

To date, the precise duration of the PICT is yet to be estimated. Ideally, the PICT should consider the pharmacokinetics of exposure to the cytotoxic agent and/or its active metabolites, which correspond to peak plasma concentrations, drug half-life, and potential interactions.^28^, ^50^ Considering patients treated with docetaxel regimens, PICTs of 90’ and 45’ had similar results preventing hair loss (79% *vs*. 95% of patients with success, respectively; *p* = 0.04).^53^ Komen et al. studied PICTs of 20’ and 45’, also in patients taking docetaxel, with comparable rates of hair retention (73% *vs*. 79% of patients with success, respectively; *p* = 0.5).^54^ In anthracycline regimens, PICTs of 90’ or greater are often used; however, prolonging PICT showed no reduction in the need for scalp coverage.^55^ Further studies are needed to determine the most effective PICT for each chemotherapy regimen.

### Hair loss assessment and scalp cooling results

The Common Terminology Criteria for Adverse Events (CTCAE) is largely used in oncology to systematize the adverse events of cancer treatment. According to the CTCAE v. 5.0, alopecia grade 1 corresponds to less than 50% of hair loss, not requiring the use of a wig or a head cover; in alopecia grade 2, hair loss is more than 50%, and a wig or a scarf is necessary.^56^ A modified Dean's scale for hair loss is also used to quantify the severity of CIA (grade 0: no hair loss; grade 1: 0 to ≤25% hair loss; grade 2: >25% to ≤50% hair loss; grade 3: >50 to ≤75% hair loss; grade 4: >75% hair loss). In the literature, scalp cooling is generally considered successful when alopecia is less than 50% (CTCAE v. 5.0 grade 0 or 1; modified Dean's scale grade 0.1 or 2).^9^, ^34^, ^57^

### Factors influencing scalp cooling success

The success of scalp cooling may vary according to the chemotherapeutic drug, dose, and combination, and also depends on the scalp temperature achieved, duration of cooling, and proper fitting of the cap.^33^, ^48^, ^58^ In the literature, there is variability in the success rates of scalp cooling for the same chemotherapy regimen and dose. Consideration should be given to the study design, population characteristics, applied technique, PCT/PICT, hair loss assessment scale, and the selected success criteria. In a Dutch registry study with 1411 patients (scalp cooling performed with Paxman devices), regimens with low-dose docetaxel (75 mg/m^2^) and paclitaxel (70–90 mg/m^2^) showed better rates of success with scalp cooling (94% and 81%, respectively) whereas among patients taking anthracycline and cyclophosphamide, 39% were successful.^33^ Rugo et al. reported success in 66.3% of patients receiving taxanes with scalp cooling with Dignitana devices.^34^ Nangia et al. reported that scalp-cooled patients using Paxman devices under taxane-based regimens are more likely to have higher rates of hair preservation (59% success; paclitaxel used weekly is more effective than docetaxel used every three weeks) than anthracycline-based chemotherapy (16% success).^9^

### Scalp cooling and cutaneous scalp skin metastases

Since the first reports of scalp cooling studies, safety concerns have been raised regarding the possibility that chemotherapy does not sufficiently treat pre-existing subclinical scalp skin metastases.^59^ The incidence of metastases in scalp-cooled breast cancer patients appears to be less than 1.1%, which is within the rates (0.03–3%) reported in breast cancer patients treated without scalp cooling.^46^, ^60^ In a study involving 442 patients using a cold cap, the overall incidence of scalp skin metastases was 0.45% (2/442), occurring in 0.88% (2/227) of breast cancer patients.^59^ Similar low rates of scalp skin metastases (7/640, 1.1%) were also noted in a retrospective cohort study of women with breast cancer, wherein the majority (86.4%) had been offered scalp cooling at some point during their treatment. It is noteworthy that in neither case did scalp metastases manifest as the first isolated site of recurrence – women who had a high risk of breast cancer recurrence, such as those in stage III, were more likely to have metastases in the scalp, in addition to other organs.^61^ Although additional research with longer follow-up is needed to establish a clear association, data available indicate that there is no increased risk of a poor outcome with scalp cooling in breast cancer patients.^7^, ^62^

However, regression of scalp skin metastases, despite scalp cooling, has also been reported (two cases), suggesting that the distribution of chemotherapy at these sites is not completely prevented.^59^, ^63^ Nevertheless, in patients with blood cancers presenting with cancer cells all over the body (*e.g*. leukemia, lymphoma), scalp cooling is currently not recommended.^46^, ^64^ Witman et al. reported a case of mycosis fungoides that disappeared with consolidation chemotherapy but recurred on the scalp.^64^ Another instance of relapse was described in a 17-year-old boy whose acute myeloblastic leukemia manifested with several subcutaneous scalp nodules but with no evidence of hematological relapse seven years after the use of scalp cooling during his two courses of chemotherapy.^65^

### Adverse events and contraindications for scalp cooling

Scalp cooling has been shown to be a well-tolerated supportive care.^49^, ^66^ The most commonly reported AEs with scalp cooling are low-grade and include headaches, nausea, dizziness, complaints of coldness, and claustrophobia. Frostbite was reported in a few cases with cold caps, which are usually below −25 °C before being applied to the patient's head; none has been reported with cooling machines.^9^, ^33^, ^50^, ^58^, ^67^, ^68^ Hairless areas (*e.g.*, forehead, ears, scalp alopecia) should be protected during scalp cooling, using a simple bandage.

Scalp cooling is not indicated for patients with cold agglutinin disease, cryoglobulinemia, cryofibrinogenemia, or cold sensitivity.^50^ It is not recommended for patients with hematologic tumors, who are at higher risk for cutaneous metastases.

### Scalp cooling and impact on quality of life and well-being

Although the use of scalp cooling has become increasingly widespread to prevent CIA, studies have shown inconsistent evidence of improvement in patients’ quality of life (QoL), reportedly due to nonspecific QoL assessments (most studies used EORTC QLQ-C30 and -BR23), differences in methods and outcomes reporting QoL and overall cost and financial burden of scalp cooling.^47^ When scalp cooling does not work as expected, the impact on QoL is worse when compared to controls, *i.e.*, patients not using scalp cooling.^33^ Therefore, an appropriate QoL instrument is still sought. In one of the first multi-centered prospective studies designed to investigate the impact on well-being in breast cancer patients receiving scalp cooling (98/266), 52% of the patients who experienced effective scalp cooling reported better well-being when compared to patients in whom this was not achieved.^69^

## Final considerations

Scalp cooling is an approved and generally well-tolerated option to prevent CIA and can minimize the burden of cancer treatment and potential impairments in patients’ health-related QoL and psychosocial well-being. While the type of scalp cooling method might not be decisive, maintaining stable low scalp skin temperatures is crucial. Patients receiving anthracycline-based regimens have lower rates of hair retention compared to taxane-based chemotherapies. Patients with hematological malignancies and cold-precipitated diseases should not undergo scalp-cooling therapy. Scalp cooling does not appear to increase the risk of cutaneous scalp metastases in early-stage breast cancer patients, nor does it appear to compromise cancer outcome, although long-term follow-up studies are needed.

## Financial support

None declared.

## Authors’ contributions

Giselle de Barros Silva: Approval of final version of the manuscript; conception and planning of the study; drafting and editing of the manuscript; collection, analysis, and interpretation of data; participation in study design; critical review of the literature; critical review of the manuscript.

Kathryn Ciccolini Hernandez: Approval of final version of the manuscript; drafting and editing of the manuscript; participation in study design; critical review of the manuscript.

Aline Donati: Approval of final version of the manuscript; critical review of the manuscript.

Corina van den Hurk: Approval of final version of the manuscript; critical review of the manuscript.

## Conflicts of interest

None declared.
